# M. Cazenave on the Different Sorts of Caustics

**Published:** 1845-09

**Authors:** 


*M. Cazenave on the different sorts of Caustics.* The *Powder of Dupuytren* is composed of one part of arsenious acid and

200 parts of calomel. It is a mild and very manageable caustic, that is useful in cases of lupus in women and children, when the ulceration is superficial and of limited extent. If the diseased part be dry, it may be necessary to denude it by means of a blister, and then to sprinkle the powder upon the raw surface. A certain amount of heat and painful swelling is usually caused by this application. When it falls off, there is generally observed a decided modification of the diseased surface. A few applications are sufficient to effect a cure in a great many instances.

The *Vienna powder and paste* are remedies of great power in certain cases of lupous ulceration. They are composed of equal parts of powdered quicklime and potassa cum calce. In using it, we take a portion of this mixture, and add a small quantity of spirits of wine to bring the powder to the consistence of a paste. A piece of adhesive plaster, with a hole in it of the size of the eschar, should be laid over the diseased surface, and the paste is then applied on the exposed parts. It is to be left for ten or twenty minutes, according to the depth of the eschar that is wished, and the ability of the patient to endure the pain.

The *chloruret of zinc paste* is much used in the present day. It is made by mixing one part of this substance with one or two parts of flour, moistening the mixture with as little water as possible. The pain produced by this application usually lasts for several hours. A greyish-colored eschar is formed; and this, in most cases, remains attached for two or three weeks before it is separated. The surface underneath is generally not ulcerated. M. Cazenave very frequently has recourse to this caustic in certain cases of lupus, to destroy the non-ulcerated tubercles.

For this purpose, he usually applies only a very thin layer of the paste, so as not to destroy the entire tubercle; and in this manner he often succeeds in effecting a complete resolution of it, without any scar being left behind.

In very many cases of long standing and deeply corroding lupous ulceration, he gives the preference to the arsenical paste over the two others which we have mentioned: its action is twofold; local as a caustic; and general by becoming absorbed, and exercising a potent alterative or modifying influence upon the economy. The following is the formula which he invariably uses:

Take of White oxyde of arsenic, 2 parts.
Sulphate of mercury, 1 part.
Animal charcoal in powder, 2 parts.

When used, a small quantity of this powder is to be made into a thin paste by the addition of a few drops of water; this is put upon the denuded surface—which should seldom or never exceed in extent that of a franc-piece. This application usually produces not only very sharp pain, but also a severe erysipelatous swelling, which lasts for 24 or 36 hours, and is sometimes accompanied with grave constitutional symptoms. Generally these subside very quickly; and then there remains on the cauterized part a hard brown crust, which often adheres for nearly a month, before it is detached.

*Fluid Caustics.*—M. Cazenave frequently makes use of a solution of the sulphate of copper for the removal of those small warts that often form upon the shoulders and back, and also of certain pediculated horny productions, which occasionally appear upon these parts. A stronger solution must be used for the latter form of cuticular excrescence.

In the treatment of favus and tinea, he recommends a weak solution either of this salt of copper, or of the nitrate of silver, or of acetic acid.

Of fluid caustics, one of the most potent and useful is the acid nitrate of mercury. When used to the surface pure and undiluted, it acts as a mere caustic; but when considerably weakened, and especially when applied to a large surface, it is unquestionably absorbed, and then it acts on the system.

It usually causes a good deal of pain and inflammatory swelling. The cases most benefitted by its application are those of lupus, in which the ulceration is extensive and not deep-seated.

The erysipelatous inflammation, which this as well as other caustics—more especially the arsenical paste—are apt to produce, need not be much dreaded; nay, the effects of the cutaneous phlegmasia seem sometimes to be decidedly salutary in the end.—*Annales des Maladies de la Peau*, Oct. 1844.

M. Gibert has recorded in a recent No. (Oct. 1844) of the Revue Medicale, a case of severe scrofulous lupus of the face, in which the progress of the disease was arrested and the extensive ulcerated surface became cicatrized under the employment, external as well as internal, of cod-liver oil. The use of this medicine was steadily persevered in for a full year and a half. During this time not only did the local malady become healed, but the general health—which had formerly been very weak and ailing—was very decidedly improved.

The patient was a young woman, and the disease had existed for nearly six years. On one occasion she had derived very considerable benefit from the internal administration of the deuto-ioduret of mercury, and the external use of the proto-ioduret ointment; but the benefit was temporary only. She had been subjected to a regular and protracted course of iodine treatment; but certainly with no advantage.—*Medico-Chiv. Rev.* in *Bulletin of Med. Science.*

